# Evaluation of sensory loss in the feet and associated factors in ambulatory patients with diabetes: a cross-sectional study

**DOI:** 10.20945/2359-4292-2025-0068

**Published:** 2025-09-24

**Authors:** Rafael Gusmão Santos Barreto, Tonnison de Oliveira Silva Filho, Ana Cláudia Rebouças Ramalho Lacerda, Breno Gabriel Araújo Sampaio de Jesus, Cícero Fidelis Lopes

**Affiliations:** 1 Faculdade de Medicina, Universidade Federal da Bahia, Salvador, BA, Brasil

**Keywords:** Diabetes mellitus, Diabetic foot, Diabetic neuropathies

## Abstract

**Objective:**

To determine the prevalence of protective sensory loss in patients with diabetes mellitus at a university hospital and to identify clinical and sociodemographic factors associated with this condition.

**Methods:**

This cross-sectional study was conducted with diabetic patients attending specialized outpatient clinics. Data were collected through patient interviews and medical record reviews, in conjunction with using the monofilament test to assess protective sensory loss in the feet. Statistical analyses included descriptive and exploratory tests, as well as bivariate and multivariate analyses to identify factors associated with sensory loss (p < 0.05).

**Results:**

A total of 184 patients were interviewed, but only 169 were included in the primary outcome analyses. The median age was 61 years, with the majority being female (72%), self-identifying as mixed-race (54%), and diagnosed with type 2 diabetes mellitus (87%). The prevalence of protective sensory loss was 20%. Factors such as a longer duration of diabetes mellitus (95%CI 1.01-1.09; p = 0.022), the presence of target organ damage (95%CI 1.25-6.84; p = 0.015), and increased body weight (OR = 1.04; 95%CI 1.01-1.07; p = 0.007) were significantly associated with sensory loss. Although systemic arterial hypertension was initially associated in the bivariate analysis, it did not remain an independent predictor.

**Conclusion:**

The significant prevalence of protective sensory loss and the lack of awareness about the monofilament test among many patients emphasize the need to expand neuropathy screening and health education in diabetes management.

## INTRODUCTION

Diabetic foot (DF) remains highly prevalent despite advances in pharmacological therapy and the early diagnosis of diabetes mellitus (DM) (^1^). It is a chronic complication of sustained hyperglycemia, characterized by a triad of peripheral neuropathy, arteriopathy, and wound infection. This combination predisposes patients to ulcers, which may progress to necrosis, gangrene, sepsis, or amputation (^2^).

Diabetic foot accounts for approximately 50 to 70% of all non-traumatic amputations (^3^). Globally, a foot or leg is amputated every 20 seconds as a result of diabetes-related complications (^4^). The negative impact on quality of life is well documented, contributing to increased morbidity and mortality, as well as to the social stigma associated with chronic, potentially disabling diseases (^5^). Beyond individual health consequences, DF imposes a substantial economic burden on public health systems (^6^).

Given this scenario, implementing public health measures focused on prevention is essential to reducing the prevalence of DF, improving patients’ quality of life, and optimizing healthcare expenditures. National and international guidelines recommend regular foot examinations to identify patients at risk of ulceration, based on evaluation of loss of protective sensation and the presence of peripheral arterial disease (^3^,^7^,^8^). Screening for peripheral neuropathy using the Semmes-Weinstein 10 g monofilament test is recommended. This method minimizes examiner-induced variability in tactile stimulation force and serves as a low-cost, highly effective tool for early diagnosis. Emphasizing the use of this test is particularly relevant, as neuropathy is often one of the earliest complications of DF, with purely neuropathic ulcers accounting for 45 to 60% of cases (^3^).

Loss of sensation in the lower limbs increases susceptibility to trauma, facilitating the development of wounds, especially when wearing inadequate footwear or walking barefoot. Beyond sensory neuropathy, motor nerve impairment leads to intrinsic foot muscle atrophy, disrupting the balance between flexor and extensor muscles, and resulting in osteoarticular deformities. These structural changes alter pressure distribution on the plantar surface, generating abnormal biomechanical loads that lead to hyperkeratosis, skin breakdown, and ulceration. The absence of pain exacerbates this process, as continued ambulation delays wound healing (^1^). Another critical factor is the association between neuropathy and angiopathy, which frequently masks symptoms of ischemic pain. This progression culminates in impaired skin integrity and barrier function, facilitating infection and worsening clinical outcomes (^1^).

Therefore, assessing sensory loss and foot care practices in diabetic patients is crucial for ensuring adherence to preventive measures and reducing the incidence of severe complications, such as ulcers and amputations. This study aimed to determine the prevalence of protective sensory loss in patients with DM at a university hospital and to identify clinical and sociodemographic factors associated with this condition. The specific objectives include estimating the prevalence of protective sensory loss, assessing awareness of screening tests, and analyzing clinical and sociodemographic factors associated with these outcomes.

## METHODS

This cross-sectional study was conducted based on data collected through direct patient interviews and medical records at a public university hospital in Northeastern Brazil. It included adult patients (aged ≥ 18 years) with a confirmed diagnosis of DM, who were receiving care at diabetes outpatient clinics from November 2023 to May 2024. As an exploratory study, no sample calculation was conducted. Patients with potential confounding factors, such as a history or active case of foot ulcers, prior lower limb amputation, or a diagnosis of any condition capable of causing peripheral polyneuropathy, were excluded. Additionally, patients who refused to sign the informed consent were excluded. Participants were recruited from two specific outpatient clinics:

The insulin therapy clinic, catering primarily to patients with type 1 DM (DM1) or insulin-requiring type 2 DM (DM2), typically involved younger individuals without established target organ damage.

The DM2 clinic focuses on older patients with pre-existing complications. Data on previous sensory testing were gathered through patient interviews to determine whether they remembered undergoing any evaluation for loss of tactile and vibratory sensation. Specifically, participants were asked about prior testing using a tuning fork, monofilament, or fishing line, as well as any other sensitivity assessments, such as touch with a pen tip or fingertip.

Sensory testing was conducted by trained examiners or a vascular surgeon with over 40 years of experience and extensive research in DF care. The Semmens-Weinstein 10 g monofilament test was used at the ten sites recommended by the University of Texas. Protective sensory loss was defined as insensitivity at four or more sites in at least one foot (^9^).

In addition to medical record review and sensory testing, a structured questionnaire was administered to collect information on demographic and clinical variables, including name, age, sex, place of birth, residence, duration of diabetes, self-reported ethnicity, weight, awareness of sensory loss screening, annual number of medical visits, and the presence of systemic arterial hypertension and dyslipidemia.

Body weight (kg) was treated as a continuous variable to facilitate a more accurate assessment of its linear association with sensory loss. Body mass index (BMI) was not calculated due to the unavailability of systematic patient height records or measurements during interviews.

Statistical analyses were undertaken using R software (R Core Team, New Zealand). Categorical variables were expressed as proportions, and quantitative variables were summarized as means (standard deviation) or medians (interquartile range). The normality of continuous variables was evaluated using the Shapiro-Wilk test and through analysis of skewness and kurtosis. Exploratory analyses examined associations between clinical and sociodemographic characteristics and two primary outcomes: protective sensory loss, characterized by abnormal monofilament test results in at least one foot; and prior sensory loss screening, determined by patient recall of previous testing (yes/no). Variables associated at p < 0.1 in bivariate analysis were included in a multivariate logistic regression model, adopting a significance threshold of p < 0.05 and a 95% confidence interval (95%CI).

This study was approved by the Research Ethics Committee of the center (approval no. 6,500,840 and CAAE no. 74843923.5.0000.0049). Data collection commenced only after obtaining ethical approval. Written informed consent was obtained from all participants before enrollment.

## RESULTS

### Characteristics of the patients

A total of 184 patients were recruited, of whom 15 were excluded from the analysis of the primary outcome because they did not undergo the monofilament test. This exclusion was due to logistical issues stemming from high patient volume in the outpatient clinic, limiting the time available for the examination before the healthcare consultation began. The sample showed a predominance of female participants (72%), with ages ranging from 21 to 79 years and a median age of 61 years (52.5-68.5). The majority of individuals self-identified as mixed-race (54%), were single (39%), and had an educational level of incomplete elementary education (41%) (**Table 1**).

**Table 1:** Basal sociodemographic characteristics of the sample

Characteristics	
Age, years	61 (52.5-68.5)
Biological sex Female	133 (72)
Ethnicity Black Brown White	79 (43) 99 (54) 5 (^3^)
Marital status Single Married Divorced Widowed	71 (39) 68 (37) 12 (^7^) 31 (^17^)
Level of education Illiterate Incomplete elementary school education Complete elementary school and incomplete high school education Complete high school and incomplete higher education Complete higher education	6 (^3^) 76 (42) 34 (19) 56 (30) 11 (^6^)

Results expressed as median (interquartile interval) or n (%).

Regarding clinical characteristics, 12.5% of patients were diagnosed with DM1, and 86.4% with DM2. The duration of diagnosis ranged from 1 to 46 years, with a median of 18 years (10 to 25). Target organ damage associated with diabetes was reported in 43% of patients. The median number of annual outpatient visits was 3 (2 to 4). The most commonly associated comorbidities were dyslipidemia (80%) and systemic arterial hypertension (63%) (**Table 2**).

**Table 2:** Clinical characteristics of the sample

Characteristics	
Type of diabetes 1 2	23 (^13^) 159 (87)
Diagnostic time, years 1-10 11-20 20-30 > 30	52 (28) 69 (38) 45 (24) 18 (^10^)
Comorbidities Dyslipidemia Systemic arterial hypertension	144 (80) 131 (72)
Target organ injury Yes	78 (43)

Results expressed as n (%).

Regarding knowledge about DF and prior screening for protective sensory loss, 52% of patients were unaware of the condition, and 53% had never undergone any foot sensitivity screening test. During the 10 g monofilament test, 34 participants (20%) presented with alterations classified as protective sensory loss.

### Sensitivity loss

The bivariate analysis of sensory loss outcomes included variables such as sex, ethnicity, education level, marital status, place of birth, residence, type of diabetes, target organ damage, diabetes duration, systemic arterial hypertension, dyslipidemia, and prior screening for protective sensory loss. Patients with pre-existing target organ damage showed a higher prevalence of sensory loss (30% *versus* 12.2%; p = 0.004). There was also a difference in the median duration of diabetes between participants with and without sensory loss (20 years [15-29] *versus* 16 years [10-25]; p = 0.023), indicating a higher probability of sensory impairment with longer diabetes duration. The association between diabetes and systemic arterial hypertension also correlated with a higher prevalence of sensory impairment (23.6% *versus* 9.1%; p = 0.038). Additionally, increased body weight was significantly associated with a higher prevalence of protective sensory loss (81 kg [67-89] *versus* 70 kg [61-79.5]; p = 0.004) (**Table 3**).

**Table 3:** Comparison between groups with and without loss of protective sensitivity

Characteristic	With loss (n = 34)	Without loss (n = 134)	p-value
Age, years	60 (55-66)	62.50 (50-68)	0.803
Weight, kg	81 (66.00-92.50)	70 (60.00-80.00)	0.004
Duration of diabetes, years	20 (^15^-30)	16 (^10^-24)	0.023
Consultations, years	3 (^2^-^4^)	3 (^2^-^4^)	0.307
Sex Female Male	22 (64.7) 12 (35.3)	99 (73.3) 36 (26.7)	0.319
Ethnicity Black Brown White	17 (51.5) 16 (48.5) 0 (0)	56 (41.5) 75 (55.6) 4 (^3^)	0.487
Level of education Illiterate Incomplete elementary school education Incomplete high school education Incomplete higher education Complete higher education	1 (^3^) 15 (45.5) 7 (21.2) 8 (24.2) 2 (6.1)	5 (3.7) 53 (39.3) 25 (18.5) 44 (32.6) 8 (5.9)	0.915
Marital status Single Married Divorced Widowed	12 (36.4) 12 (36.4) 2 (6.1) 7 (21.2)	55 (41) 50 (37.3) 9 (6.7) 20 (14.9)	0.843
Target organ injury	21 (63.6)	49 (36.3)	0.004
Systemic arterial hypertension	29 (87.9)	94 (70.1)	0.038
Dyslipidemia	28 (87.5)	106 (79.7)	0.310

Results expressed as median (interquartile interval) or n (%).

In the multivariate analysis, target organ damage remained significantly associated with sensory loss, yielding an odds ratio (OR) of 2.86 (95%CI 1.25-6.84; p = 0.015). Similarly, diabetes duration also maintained a statistically relevant association, with an OR of 1.05 (95%CI 1.01-1.09; p = 0.022), suggesting that each additional year of diagnosis increases the likelihood of losing protective sensation. Body weight, analyzed as a continuous variable, was also associated with sensory loss (OR = 1.04; 95%CI 1.01-1.07; p = 0.007), indicating that weight gain contributes to a higher prevalence of neuropathy. However, systemic arterial hypertension did not maintain a statistically significant association with sensory loss after adjusting for other variables (OR = 1.32; 95%CI 0.42-5.03; p = 0.657) (**Table 4**).

**Table 4:** Multivariate logistic regression model for the prediction of loss of protective sensitivity in individuals with diabetes mellitus

Variable	OR (95%CI)	p-value
Weight	1.04 (1.01-1.07)	0.007
Duration of diabetes	1.05 (1.00-1.09)	0.022
Target organ injury	2.86 (1.25-6.84)	0.015
Systemic arterial hypertension	1.32 (0.42-5.03)	0.657

OR: odds ratio; 95%CI: 95% confidence interval.

### Prior screening

Regarding prior screening for sensory loss, a higher prevalence of testing was observed in individuals with systemic arterial hypertension (52.7% *versus* 34.7%; p = 0.032) and dyslipidemia (53.5% *versus* 25.7%; *p* = 0.003). Similarly, those with a longer median duration of diabetes (20 [15-30] *versus* 13 [8-20]; p < 0.001) also showed a higher frequency of screening. The variable “target organ damage” was associated with prior screening for sensory loss as well. This association, however, was inverse compared to the other variables; 60.5% of patients with target organ damage reported never having undergone sensitivity testing, compared to 47.1% of patients without target organ damage (p = 0.075).

In the multivariate analysis, target organ damage, dyslipidemia, and the duration of diabetes remained significantly associated with prior screening for protective sensory loss. Target organ damage was inversely associated with a 62.4% lower likelihood of screening among patients with this condition (OR = 0.376; 95%CI 0.183-0.750; p = 0.006), reinforcing the theory that target organ damage correlates with a lower prevalence of sensory loss screening.

Conversely, patients with dyslipidemia had a higher likelihood of having undergone sensitivity testing (OR = 2.868; 95%CI 1.142-7.696; p = 0.029). The duration of diabetes was positively associated with prior screening, with a 7.3% increase in the likelihood of testing for each additional year since diagnosis (OR = 1.073; 95%CI 1.036-1.113; p < 0.001). However, systemic arterial hypertension did not maintain a significant association with sensitivity testing (p = 0.370) (**Table 5**).

**Table 5:** Multivariate logistic regression model for the prediction of submission to protective sensitivity loss test in individuals with diabetes mellitus

Variable	OR (95%CI)	p-value
Duration of diabetes	1.07 (1.04-1.11)	<0.001
Target organ injury	0.38 (0.18-0.75)	0.006
Dyslipidemia	2.87 (1.14-7.70)	0.029
Systemic arterial hypertension	1.45 (0.64-3.32)	0.370

OR: odds ratio; 95%CI 95% confidence interval.

## DISCUSSION

This study assessed 184 patients with DM at a specialized outpatient clinic, 169 of whom completed the monofilament test. We observed a significant prevalence of protective sensory loss in 34 patients (20% of the sample). According to the literature, the average prevalence of peripheral neuropathy ranges from 28 to 50%, depending on the criteria used, while Brazilian studies have reported a prevalence of 16 to 37% (^10^-^16^). These variations may result from differences in assessment methods, such as the number of monofilament application sites, the inclusion of additional tests, and the clinical profiles of the patients, especially in terms of comorbidities (^10^-^13^). Our findings highlight a substantial risk of neuropathy, underscoring the importance of preventive measures and screening.

The relationship between dyslipidemia and protective sensory loss involves multiple patho-physiological mechanisms. Elevated lipid levels and increased adiposity contribute to chronic low-grade systemic inflammation, leading to the release of pro-inflammatory cytokines such as tumor necrosis factor-alpha (TNF-α) and interleukin (IL) 6, which can damage peripheral nerves. Additionally, dyslipidemia and obesity may cause endothelial dysfunction and microvascular impairment, limiting nerve perfusion and resulting in ischemic injury. Furthermore, lipid accumulation within peripheral nerves (lipotoxicity) can heighten oxidative stress and mitochondrial dysfunction, further compromising nerve integrity (^14^-^16^).

In our study, body weight was another significant variable associated with protective sensory loss in the multivariate analysis. This finding aligns with existing literature that suggests excess weight exacerbates neuropathy by promoting systemic inflammation and vascular changes that impair nerve integrity (^17^). Brinati and cols. (^16^) identified elevated BMI as a primary predictor of sensory loss in patients with DM2, highlighting the necessity of weight management strategies in minimizing neuropathy risk. Therefore, in addition to glycemic and lipid control, weight management should be considered a crucial therapeutic (^16^).

Diabetes duration also showed a positive correlation with sensory loss, with patients having over 10 years of disease progression at a significantly higher risk for neuropathy. This increment in prevalence with disease duration mirrors findings from other studies. For instance, Young and cols. (^10^) reported a prevalence of 36.8% among patients with over 15 years of DM, while Silva and cols. (^11^) noted that neuropathy becomes increasingly prevalent after the tenth year of diagnosis, particularly among those with inadequate glycemic control. This temporal link between neuropathic damage and disease duration can be attributed to the cumulative effects of glycemic insult, which intensifies with prolonged exposure to hyperglycemia and chronic inflammation (^10^,^11^).

Although initial bivariate analysis indicated that systemic arterial hypertension might be a risk factor for sensory loss, subsequent multivariate analysis did not confirm this association. This inconsistency might stem from the cross-sectional study design and the adjustment for confounding factors such as dyslipidemia and disease duration in the analysis, which could have diminished the perceived impact of hypertension. Dutra and cols. (^12^) found a statistically significant association between hypertension and neuropathy only in patients with additional comorbidities, indicating that isolated hypertension may not sufficiently explain protective sensory loss.

Regarding preventive screening, 53% of patients reported never having undergone sensitivity testing, which unveils gaps in preventive screening practices among the population studied. This finding is alarming, considering the national and international guidelines that recommend annual foot evaluations for all diabetic patients, irrespective of disease duration (^7^,^18^). The infrequency of testing underscores the need for enhanced training for healthcare professionals and the integration of neuropathy assessment protocols into routine care more effectively. Additionally, memory bias might play a role, as patients could fail to remember undergoing the test, especially if foot evaluations are conducted informally (^17^).

When analyzing the prevalence of sensitivity testing, variables such as the DM duration, dyslipidemia, and elevated blood pressure were found to be associated with a higher likelihood of undergoing screening. Patients with a longer duration of disease and comorbidities, including dyslipidemia and hypertension, are often perceived by physicians as higher-risk, warranting closer monitoring. Consequently, these patients are more likely to undergo preventive strategies, such as protective sensory testing. The presence of dyslipidemia, for instance, suggests an increased risk of microvascular complications, leading to intensified neuropathy screening, especially in patients with long-standing disease. Similarly, hypertension may act as a risk marker, indicating the need for stricter monitoring to prevent further complications.

An intriguing and clinically relevant finding of this study was the inverse association between the presence of target organ damage and the likelihood of receiving sensory screening with the monofilament test. A plausible explanation is what clinical cognitive psychology identifies as anchoring bias (^17^). In busy outpatient settings, particularly in public health systems facing high patient volumes and time constraints, healthcare professionals may unconsciously prioritize managing diagnosed and overt complications (e.g., stroke, coronary artery disease, or advanced nephropathy) over screening for latent or subclinical conditions such as peripheral neuropathy. This may result in a “clinical anchor” that disproportionately directs the physician’s attention and resources, narrowing the scope of preventive assessment and fragmenting comprehensive care.

This phenomenon may also be reinforced by the perception (explicit or implicit) that once chronic complications have occurred, preventive screening may have limited utility, leading to pragmatic but flawed clinical shortcuts. This is particularly concerning in diabetic neuropathy, which is both progressive and modifiable if detected early. Paradoxically, patients at highest risk may be the least likely to be systematically screened. Our findings underline the critical need to implement structured protocols and standardized checklists in chronic care workflows, ensuring that preventive measures, such as the monofilament test, are not overlooked, even amid more urgent or complex clinical demands. The cross-sectional study design limits the ability to establish definitive causal relationships.

One limitation of this study was the lower median age of DM1 patients (35 years; interquartile range [IQR] of 23-47) compared to DM2 patients (63 years; IQR of 54-69), which prevented a thorough evaluation of the influence of diabetes type on protective sensory loss. Younger patients exhibit peripheral neuropathy less prevalently, potentially underestimating its contribution to this complication (^3^). Future studies should include a larger number of DM1 patients with a longer disease duration to better explore differences between diabetes types.

This study also faced other methodological limitations. As a single-center study using a convenience sample, its results may not be generalizable to other populations. Additionally, the cross-sectional design precludes the inference of causal relationships, and the exploratory statistical analysis may have diminished the robustness of the observed associations. Additionally, the exclusion of 15 patients due to logistical reasons might have introduced selection bias, although this loss was not directly related to the patients’ clinical characteristics, but to the dynamics of a high-volume outpatient clinic. Similarly, BMI could not be assessed, as measuring patient height was impractical in this setting, which may partially limit the findings’ generalizability.

Despite these limitations, this study presents noteworthy strengths. It suggests potential inefficiencies in diabetes care, emphasizing the need for improved coordination among healthcare professionals and health networks for proper DF screening and management. With a sample of 184 patients, larger than similar national studies, and data collection conducted by a single researcher group using a standardized monofilament, data variability was minimized, enhancing the precision of the findings. This study also emphasized the importance of managing comorbidities such as dyslipidemia and weight, alongside strict glycemic control and monitoring disease duration, to mitigate neuropathic complications. The findings align with existing literature and underscore the significance of promoting health education and early screening strategies to prevent severe complications.

More notably, patients with active foot ulcers, a history of previous ulcers, or prior lower limb amputations were excluded based on predefined eligibility criteria to assess protective sensory loss in individuals without overt clinical complications. Including patients with advanced DF manifestations could have introduced selection bias, as they are more likely to undergo systematic screening due to the severity of their condition. By focusing on patients without visible foot complications, we sought to capture subclinical neuropathy and identify gaps in screening practices early, highlighting opportunities for intervention.

An additional strength was the educational intervention post-data collection. The 184 participants received guidance on DF care, the importance of screening, preventive measures, and potential complications of neuropathy. Although descriptive, this approach added a vital dimension by raising awareness and reinforcing comprehensive patient care in line with best clinical practices.

This study’s findings regarding the prevalence of protective sensory loss among diabetic patients indicate a significant risk of neuropathic complications, emphasizing the importance of screening. Many patients, even those with a long-standing diagnosis or existing complications, were unaware of the monofilament test and reported never having undergone sensitivity assessment, underscoring the need for broader and more accessible implementation of the monofilament test in clinical practice to enhance awareness among both patients and healthcare professionals about the importance of neuropathy screening in diabetes management.

Our findings reinforce the urgency of systematic neuropathy screening in primary care settings to improve early detection, guide timely interventions, and reduce complications such as foot ulcers and amputations. Therefore, this study highlights the critical need for educational strategies and continuous foot health monitoring in diabetic patients to prevent the progression of neuropathic lesions and enhance their quality of life.

### Perspectives and clinical recommendations

This study emphasizes the importance of incorporating structured foot screenings, particularly the 10 g monofilament test, into diabetes care at all levels of healthcare. To address prevention gaps, particularly among patients experiencing complications, it is critical to establish standardized protocols, provide professional training, and engage in patient education. A comprehensive approach ought to evaluate not only comorbidities but also subclinical complications, such as peripheral neuropathy. The early identification of such issues allows for prompt interventions, thereby mitigating severe outcomes. Further research, through longitudinal studies, is necessary to determine if more extensive screening can reduce the incidence of neuropathy and enhance patient outcomes. Expanding primary care screenings and adopting multidisciplinary strategies are crucial steps towards improving prevention efforts and alleviating public health burdens.

In conclusion, the prevalence of protective sensory loss among diabetic patients in our study underscores the importance of systematic screening with the 10 g monofilament test. Many patients, even with long-standing diabetes or established complications, reported never having undergone sensitivity testing, which highlights significant gaps in preventive care. Notably, patients with established target organ damage were less likely to be screened, suggesting a paradoxical gap in preventive practices among those at higher risk. Expanding routine neuropathy screening and patient education is crucial to reduce complications such as ulcers and amputations and to improve long-term outcomes in diabetes management. Further multicenter and longitudinal studies are needed to confirm these findings and to better define strategies for the early detection and prevention of diabetic neuropathy.

## Data Availability

datasets related to this article will be available upon request to the corresponding author.
